# Nutraceutic Potential of Two *Allium* Species and Their Distinctive Organosulfur Compounds: A Multi-Assay Evaluation

**DOI:** 10.3390/foods8060222

**Published:** 2019-06-21

**Authors:** Zahira Fernández-Bedmar, Sebastián Demyda-Peyrás, Tania Merinas-Amo, Mercedes del Río-Celestino

**Affiliations:** 1 Department of Genetics, University of Córdoba, Campus Rabanales, Gregor Mendel Building, 14071 Córdoba, Spain; tania.meram@gmail.com; 2 Institute of Veterinary Genetics (IGEVET), Facultad de Ciencias Veterinarias, UNLP-CONICET, Universidad Nacional de La Plata, La Plata 1900, Argentina; sdemyda@igevet.gob.ar; 3 Agrifood Laboratory, CAPDER, Avda. Menéndez Pidal s/n, 14004 Córdoba, Spain; mercedes.rio.celestino@juntadeandalucia.es

**Keywords:** garlic, onion, antigenotoxicity, longevity, cytotoxicity, comet assay

## Abstract

This study aimed to evaluate the biological activities of two *Allium* species (garlic and onion) as well as diallyl disulphide (DADS) and dipropyl disulphide (DPDS) as their representative bioactive compounds in a multi-assay experimental design. The genotoxic, antigenotoxic, and lifespan effects of garlic, onion, DADS, and DPDS were checked in *Drosophila melanogaster* and their cytotoxic, pro-apoptotic, and DNA-clastogenic activities were analyzed using HL60 tumoral cells. All compounds were non-genotoxic and antigenotoxic against H_2_O_2_-induced DNA damage with a positive dose-response effect and different inhibition percentages (the highest value: 95% for DADS) at all tested concentrations. Daily intake of *Allium* vegetables, DADS, or DPDS had no positive effects on flies’ lifespan and health span. Garlic and DADS exerted the highest cytotoxic effects in a positive dose-dependent manner. Garlic and DADS exerted a DNA-internucleosomal fragmentation as an index of induced proapoptotic activity on HL60 cells. *Allium* vegetables and DADS were able to induce clastogenic strand breaks in the DNA of HL60 cells. This study showed the genomic safety of the assayed substances and their protective genetic effects against the hydrogen peroxide genotoxine. Long-term treatments during the whole life of the *Drosophila* genetic model were beneficial only at low-median concentrations. The chemo-preventive activity of garlic could be associated with its distinctive organosulfur DADS. We suggest that supplementary studies are needed to clarify the cell death pathway against garlic and DADS.

## 1. Introduction

The Mediterranean diet is one of the best nutritional patterns for humans due to its demonstrated beneficial effects on health. This diet, which is based on the high consumption of fruit, vegetables, wine, olive oil, and fish as the main animal protein contribution, is a type of a healthy and well-balanced food intake [1]. Today, most of the studies asserting these well-being effects agree to point the increased antioxidant and phenolic contents as the cause of its properties [2]. Diet-derived antioxidants are implicated in maintaining a balanced homeostasis and scavenging reactive oxygen species (ROS) as a major part of a highly efficient defensive biological network, which neutralizes the oxidative stress and complements the endogenous defense enzymes [3].

Garlic (*Allium sativum*) and onion (*Allium cepa*) are two native vegetables from Asia and are widely used in different gastronomic cultures and traditional medicines for centuries [4]. According to the Food Administration Organization (FAO), these vegetables are two of the most important crops worldwide with a production of 20,000 tons of garlic and 100,000 tons of onion, respectively, in 2015, which shows a trend toward an increased consumption in the recent years due to the expansion of the Mediterranean and Asian cuisine. Both volatile and non-volatile compounds are found in Allium species. Non-volatile compounds named sapogenins, saponins, and flavonoids whose contents are differentially distributed in garlic and onions. The distinctive flavonoids present in onions are different than in garlic (quercetin, kaempferol, and luteolin in onion and myricetin, apigenin and quercetin in garlic) [5]. Quercetin, which is the major flavonoid present in onions, helps prevent glycation of collagens, which is a leading causative factor for the development of cardiovascular complication in diabetic patients. Moreover, quercetin and kaempferol from onions also possess anticarcinogenic properties [6,7]. With regard to the phenolic acids present in the matrix of garlic and onions, gallic acid is one of them and it has several reported bioactivities such as antineoplastic, bacteriostatic, antioxidant, and anticancer. Protocatechuic acid is found in these vegetables as well. This molecule has been found to have an antihepatotoxic, anti-inflammatory, free radical scavenger, including chemopreventive and apoptotic bioactivities among others [8,9]. Nevertheless, despite the above described differences between garlic and onion non-volatile content, these two species contain a unique and distinctive group of volatile organosulfur compounds.

These vegetables have been linked to preventive effects against several diseases such as cancer, obesity, diabetes type-2, coronary heart disease, and hypertension, among others [5,10,11,12]. These pleiotropic effects were associated with the high content of thiosulfinates, which is a group of volatile organosulfur compounds that originated from the decomposition of the allicin. These are also responsible for their typical pungent aroma and taste [7,13,14,15]. However, both vegetables showed a high variability with respect to the thiosulfinate profiles among strains including diallyl sulfide (DAS), diallyl disulfide (DADS), and diallyl trisulfide (DATS) normally higher in garlics and dipropyl sulfide (DPS) and dipropyl disulfide (DPDS) higher in onions [16,17]. Garlic oils and extracts were associated with several health-benefit activities, such as a protective capacity against DNA damage induced by oxidative stress, increased hydrogen peroxide (H_2_O_2_) scavenging activity, and ability to reduce the bioactivity of carcinogens and tumor cell proliferation [18,19,20,21]. These capacities were directly linked to DADS, one of their major and most garlic distinctive constituents, which was widely studied and characterized as non-genotoxic, antigenotoxic, inhibitor of cell proliferation and pro-apoptotic in different cancer cell lines like leukemia, colon, prostate, lung, bladder, and skin [22,23,24,25,26,27,28,29].

On the other hand, onions are more versatile vegetables that can also be consumed as fresh and processed products. Both forms also showed a high oxy-radical scavenging capacity [30] as well as an antigenotoxic effect [31]. In addition, garlic ethanolic extracts and oils showed antimutagenic activity [32] and also decreased the viability and increased the apoptosis in several cancer cell lines like HL60, MDA-MB-231, A549, and B16F10 [33,34,35,36]. In this case, their pro-healthy properties were widely related to DPDS, which is one of its most representative organosulfur compounds. This molecule was previously associated with strong anticarcinogenic activity [37] and a protective effect against a DNA strand break and oxidative damage [38,39]. Nevertheless, this compound had no anti-tumor effects in mice [40], which means it did not decrease tumor cell growth and did not induce DNA-internucleosomal fragments on cancer cell lines by acting alone [29,40,41,42].

Then, we performed a qualitative and quantitative evaluation of the health-beneficial properties of garlic, onion, and their representative organosulfur compounds (DADS and DPDS) in a multi-assay experimental design using in vivo and in vitro models. We assessed their genotoxic, antigenotoxic, and lifespan effects in *Drosophila melanogaster* flies, which is a widely used experimental model closely related to humans. Additionally, we evaluated their proapoptotic capacities against cancer processes through the determination of their cytotoxic, clastogenic, and DNA epigenetic modulator activity against in an in vitro human cancer model (HL60 cell line).

## 2. Materials and Methods

### 2.1. Allium Vegetables and Single Compounds

Two *Allium* species and two of its most distinctive organosulfur compounds were assayed. Garlic (*Allium sativum,* purple variety) and onion (*Allium cepa,* Victoria variety) were purchased in a local market of Cordoba (Spain). Thiosulfinates, DADS from garlic, and DPDS from onions, which had 80% and ≥97% of purity, respectively, were purchased from Sigma (St. Louis, MI, USA, Cat numbers 317691 and 43550, respectively) and were used without further purification.

### 2.2. Preparation of the Samples

Garlic samples and onions were washed twice with distilled water, cut in slim slices, and freeze-dried at −80 °C. After that, both samples were lyophilized, pulverized with a mortar pestle, sieved, and stored at 25 °C in the dark until use.

### 2.3. In Vivo Assays

#### 2.3.1. Somatic Mutation and Recombination Test (SMART)

Two *Drosophila melanogaster* strains carrying visible wing genetic markers were used in our experimental design: the flare (flr) strain flr^3^/ln (3LR) TM3, Bd^s^ and the multiple wing-hair (mwh) strain mwh/mwh. The multiple wing hairs (mwh, 3_0.3) marker is a recessive viable mutation in homozygous flies, which produces multiple-hairs trichomes in the fly adult body [43]. The flare (flr^3^, 3_38.3) marker is a homozygous recessive lethal mutation, which produces malformed individual wing hairs in somatic cells of larvae. The flr^3^ allele is retained in a balancer chromosome carrying multiple inversions and a homozygous lethal dominant visible marker expressed in the edge wing [44].

Genotoxicity was determined using the SMART test as described by Graf and Wurgler [45] including a negative control of pure water. The antigenotoxic activity was also determined using a modified SMART test following our standard protocols [46]. Optimally virgin flr^3^/ln (3LR) TM3, ri p^p^ sep bx^34e^ e^s^ Bd^S^ (flare) females were crossed with mwh/mwh strain males, obtaining 72 h transheterozygous F1 larvae after an 8-hour egg-laying on fresh yeast. Larvae were fed with *Drosophila* Instant Medium (Formula 4-24, Carolina Biological Supply, Burlington, NC, USA) in 4 mL vials. Genotoxicity assays consisted of eight experimental groups by supplementing the base larvae food (0.85 g) with different concentrations of onion (0.625 and 5 mg/mL), garlic (0.625 and 5 mg/mL), DADS (4 mM and 34 mM), and DPDS (4 mM and 33 mM). The concentration ranges of single compounds were selected to mimic those described in the fresh *Allium* sp. and they cover the lower and higher estimated content values [47]. Negative (distilled water) and positive (0.12 M H_2_O_2_) concurrent controls were included. Antigenotoxicity experimental design was similar to the genotoxicity assays by concurrently treating the larvae with the tested substances supplemented with H_2_O_2_ (0.12 M) as a positive geno-toxicant control. The emerged adults in each group were stored in 70% ethanol until analysis.

Forty wings of heterozygous flies (mwh/flr^3^) treated with each compound and concentration were removed and mounted on slides with Faure’s solution (Arabic gum 50 g (Sigma, Cat Number G9752), glycerol of 20 mL (Sigma, Cat Number G5516), chloral hydrate of 50 g (Sigma, Cat Number C8383), and distilled water of 50 mL). Both dorsal and ventral surfaces were screened under a bright light microscope at 400× magnification to detect small single spots (1–2 mwh or flr^3^ cells), large single spots (three or more cells), and twin spots (adjacent mwh and flr^3^ cells). Single spots are produced by gene mutation, somatic recombination, and deletion between the two markers. Twin spots are produced uniquely by recombination between the flr^3^ marker and the centromere.

In order to evaluate the possible genotoxic effect, the frequencies of total spots per wing of each series were statistically compared with the total spots of the negative control with the non-parametric U-test of Mann, Whitney, and Wilcoxon [48]. Antigenotoxicity was determined as the inhibition percentage (IP) using the total spots per wing determined at each concentration with the following formula [49].

IP = ((*a* − *b*)/*a*) × 100, (1)

where *a* represents the frequency of total spots induced by the treatment with genotoxine alone, and *b* represents the frequency of total spots obtained with genotoxine plus substance tested in the different combined treatments.

#### 2.3.2. Longevity Assays

All the longevity experiments were performed following our standard procedures [50]. Transheterozygous larvae from a 12-h egg-laying with the same genetic background described above were used in the life and health-span trials. Health span is the healthy adult period of unimpaired life that precedes functional decline [51]. It is important to consider the quality of a prolonged life and, for this reason, health span is a new focus in aging research. Synchronized larvae of 72 ± 12 h were clustered in groups of 100 individuals in glass vials with 0.85 g of *Drosophila* Instant Medium in 4 mL of water solutions of the different experimental concentrations assayed (0.625, 1.25, 2.5, and 5 mg/mL for *Allium* vegetables, 4, 8, 16, and 33 mM for DPDS, and 4, 8, 17, and 34 mM for DADS). The emerged flies were anesthetized under CO_2_, separated into 10 single-sex groups, transferred to longevity vials and fed with the same treated medium during the whole experimental design. A concurrent treatment was also included using distilled water as a negative control. The survivors were counted and the medium was renewed twice a week until all individuals die. Survival curves were plotted as estimated by the Kaplan-Meier method and the statistical significance of curves were assessed using the Log-Rank (Mantel-Cox) method using the SPSS 15.0 statistics software (SPSS Inc. Headquarters, Chicago, IL, USA).

### 2.4. In Vitro Assays

#### 2.4.1. Cell Line Cultures and Cytotoxicity Assay

In vitro assays were performed using the promyelocytic leukemia HL60 cell line. Some of the genetic characteristics of this tumor cell line are the following: karyotypic abnormalities (monosomy, trisomy, and tetrasomy), and different chromosomal translocations. On the molecular genetic level, the HL60 cell line has deletions in the p53 gene on chromosome 17pl3 and one allele of the GM-CSF gene on chromosome 5q21–q23 is rearranged and partly deleted as well [52].

Cells were cultured at 2.5 × 10^5^ cells/mL following our standard protocol [53] in complete RPMI 1640 medium (BioWhittaker, Basel, Switzerland; BE12-167F) containing 10% heat-inactivated fetal bovine serum (BioWhittaker, de14-801F), l-glutamine 200 mM (Sigma, G7513), and antibiotic-antimycotic solution (Sigma, A5955) at 37 °C in a humidified atmosphere of 5% CO_2_. Two passes per week were performed and the experiments were carried with cells with no more than 20 passes. Cell viability was evaluated by the Trypan blue exclusion assay. To ensure the proper behavior of the cell line, proliferation was followed at 0, 4, 24, 48, and 72 h checkpoints. Control cells doubled every 24 four-hour exponentially (*y* = 100446e^0.0345*x*^), which reached the maximum at 72 h. Cells (1 × 10^5^ cells/mL) were seeded and incubated for 72 h in 96 well plates supplemented with six different concentrations *Allium species* (ranging from 0.002 mg/mL to 0.06 mg/mL) and 6 different concentrations of thiosulfinates (ranging from 0.012 mM to 0.4 mM). A concurrent negative control (base medium without supplementation) was also run. After incubation, Trypan blue was added to the cell suspension (1:1 ratio) and cells were counted in a Neubauer chamber under an inverted microscope at 100× magnification. Cell viability was expressed as a percentage of survival with respect to control after a 72-h period. IC_50_ values (concentration of tested molecule causing 50% of cell growth inhibition) and EC_50_ values (concentration of a tested substance that complements a system and gives a half-maximal growth response) were estimated for each treatment. Viability curves were plotted as mean viability ± standard deviation of three independent replicas in each substance and concentration.

#### 2.4.2. Inter-Nucleosomal DNA Fragmentation Assay

HL60 cells (1.5 × 10^6^ cells/mL) were incubated with the same compounds and concentrations as in cytotoxicity assays for 5 h in 12-well plates. Thereafter, cells were harvested, centrifuged at 2500 rpm. for 5 min, and washed with phosphate buffer saline (PBS). Total DNA was extracted using a commercial DNA-extraction kit (Blood Genomic DNA Extraction Mini Spin Kit, Canvax Biotech, Cordoba, Spain), according to the manufacturer’s instructions and subsequently treated with RNase overnight in order to eliminate a false positive. DNA yielding was quantified in a Nanodrop™ (Thermo Scientific, Madrid, Spain). A total of 1.5 µg of DNA per sample was electrophoresed in a 2% agarose gel, stained with ethidium bromide, and run by 120 m at 60 V. Internucleosomal DNA fragmentation was determined by the presence of ladder band patterns with 200 bp multiple fragments.

#### 2.4.3. Evaluation of DNA Breakage Ability: Comet Assay

DNA strand break ability of the compounds was determined by the alkaline comet assay, as described Olive and Banáth [54] with minor modifications. HL60 cells (5 × 10^5^ cells) were plated in 1.5 mL of culture medium supplemented with different concentrations of onion (0.004, 0.016, and 0.06 mg/mL), garlic (0.002, 0.004, and 0.008 mg/mL), DPDS (0.025, 0.1, and 0.4 mM) and DADS (0.01, 0.025, and 0.05 mM) and incubated for 5 h. After treatment, cells were washed and adjusted to 6.25 × 10^4^ cells/mL in PBS. Then, cells (1.6 × 10^4^) were suspended in a 75 µL pre-warmed low melting point agarose (A4018, Sigma) and 50 µL of the suspension were rapidly spread on microscope slides and covered with coverslips. After gelling for 30 min at RT, the coverslips was gently removed and the slides were put in a tank filled with lysis solution (2.5M NaCl (S3014, Sigma), 100mM Na-EDTA (1.09992, Sigma), 10mM Tris (T4661, Sigma), 250mM NaOH (S8045, Sigma), 10% DMSO (D8418, Sigma), and 1% Triton X-100 (T8787, Sigma), pH = 13 at 4 °C for 1 h. Next, slides were removed from the lysis solution and incubated in alkaline electrophoresis buffer (300 mM NaOH (S8045, Sigma) and 1 mM Na-EDTA (1.09992, Sigma), pH = 13 at 4 °C for 20 to 30 min. Electrophoresis was then carried out in a fresh-made electrophoresis buffer for 15 min at 20 V and 400 mA in dark conditions. After electrophoresis, slices were gently washed in cold fresh-made neutralization buffer (0.4 M Tris-HCl buffer, pH 7.5) for 10 min and allowed to dry overnight at RT in dark conditions. Lastly, gels were stained with 7 µL propidium iodide (S7109, Sigma), covered with a coverslip, and photographed at 400× magnification in a Leica DM2500 epifluorescence microscope with a microscope. At least 50 cells were assessed for each treatment. Data were analyzed using the Open CometTM software [55]. The statistical ANOVA-Tukey test was applied [56] using the SPSS 15.0 statistics software (SPSS Inc. Headquarters, Chicago, IL, USA) in order to compare the results obtained for the different treatments and the negative control.

#### 2.4.4. Epigenetic Analysis of Repetitive Sequences on DNA of HL60 Cells

HL60 cells were plated and treated with two concentrations of Allium species (0.002 and 0.06 mg/mL) and two concentrations of thiosulfinates (0.012 and 0.4 mM) for 5 h. Genomic DNA from HL60 cells was isolated in the same way as described in the DNA fragmentation section. After that, Bisulphite-modified DNA from food coloring treatments (EZ DNA Methylation-Gold™Kit) was used as a template for fluorescence-based real-time quantitative Methylation-Specific PCR (qMSP). A qMSP were carried out according to the protocol described by Merinas-Amo et al. [57] in 48 well plates in the MiniOpticon Real-Time PCR System (MJ Mini Personal Thermal Cycler, Bio-Rad) and was analyzed by Bio-Rad CFX Manager 3.1 Software. The final reaction mixture (V = 10 µL) consisted of: 1 µL of bisulfite converted genomic DNA, 2 µL of milliQ water, 5 µM of each forward and reverse primer, 2 µL of iTaq™ Universal SYBR^®^ GreenSupermix (Bio-Rad, which contained antibody-mediated hot-start iTaqDNA polymerase, dNTPs, MgCl2, SYBR^®^ Green I dye, enhancers, stabilizers, and a blend of passive reference dyes including ROX and fluorescein).

qMSP conditions included initial denaturalization at 95 °C for 3 min and amplification, which consisted of 45 cycles at 95 °C for 10 s, 60 °C for 15 s, and 72 °C for 15 s, taking a picture at the end of each elongation cycle. After that, the melting curve was determined by increasing 0.5 °C each 0.05 s from 60 °C to 95 °C and taking pictures.

Repetitive elements were selected in order to analyze a wide range of human genomic DNA. While Alu and LINE sequences are interspersed throughout the genome, satellites are confined to the centromere areas [58,59,60,61]. Alu M1, LINE-1, and Sat-α sequences were used and the housekeeping Alu-C4 was used as a reference to correct for total DNA input. All primers were obtained from Isogen Life Science and their sequences are as follows: Alu-C4 (forward: 5´-GGTTAGGTATAGTGGTTTATATTTGTAATTTTAGTA-3´; reverse: 5´-ATTAACTAAACTAATCTTAAACTCCTAACCTCA-3´), Alu-M1 (forward: 5´-ATTATGTTAGTTAGGATGGTTTCGATTTT-3´; reverse: 5´-CAATCGACCGAACGCGA-3´); LINE-1 (forward: 5´-GGACGTATTTGGAAAATCGGG-3´; reverse: 5´-AATCTCGCGATACGCCGTT-3´); Sat-α (forward: 5´-TGATGGAGTATTTTTAAAATATACGTTTTGTAGT-3´. For detailed information on the primers, see Weisenber et al. [62].

The relative yielded results were normalized with the housekeeping sequence Alu C4 using the Nikoliaidis et al. [63] and the Liloglou et al. [64] comparative C_T_ method.

- C_T_ of the target gene was normalized with respect to the referent gene (ΔC_T_).
- ΔC_T_ of each experimental sample or reference (ΔC_T,r_) were compared with ΔC_T_ of the calibrator sample (ΔC_T,cb_): ΔΔC_T._
- The relative value of each sample is defined by the formula below.

2^−(ΔC_T,r_ − ΔC_T,cb_)^ = 2^−ΔΔC_T_^

Each sample was analyzed in triplicate. One-way ANOVA and post hoc Tukey’s tests were used to evaluate the differences among the tested compound, repetitive elements, and concentrations.

## 3. Results

### 3.1. SMART Test

**Figure 1:**
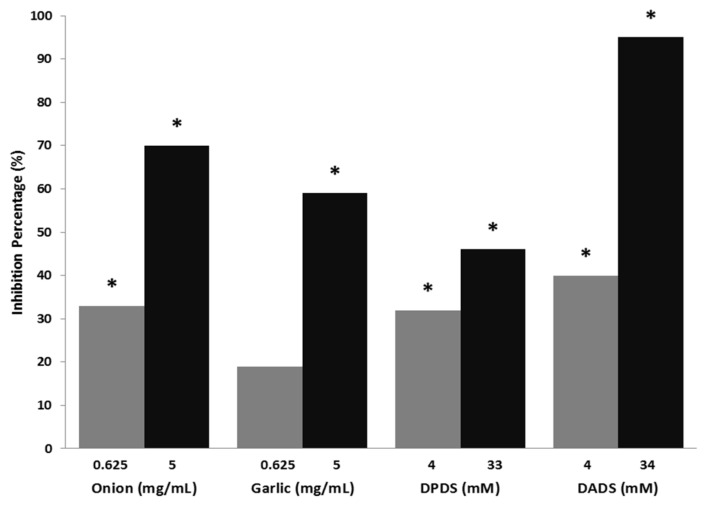
Mutagenicity inhibition percentages produced by onion, garlic, dipropyl disulphide (DPDS), and diallyl disulphide (DADS) against H_2_O_2_ – DNA induced damage (*Drosophila melanogaster* model). *: Statitiscal significance compared with the positive control using the Kastenbaum-Bowman binomial test with significance levels α = β = 0.05.

The results of genotoxicity and antigenotoxicity are shown in Table 1. All the assayed compounds were non-genotoxic in the flies at all tested concentrations. Both *Allium* vegetables showed no differences compared with water control in single and total spots. Validation of the experimental design was assessed by the results of the positive control (H_2_O_2_, 0.37 total spots/wing), which agreed with our previous results [50,65]. The antigenotoxic potency of *Allium* sp. vegetables, DPDS and DADS against H_2_O_2_ exhibited a clear positive dose-response effect even though the lowest concentration of garlic was not statistically different with respect to the positive control (Figure 1), which shows the DADS the highest IP value (95%).

### 3.2. Longevity Assays

**Figure 2:**
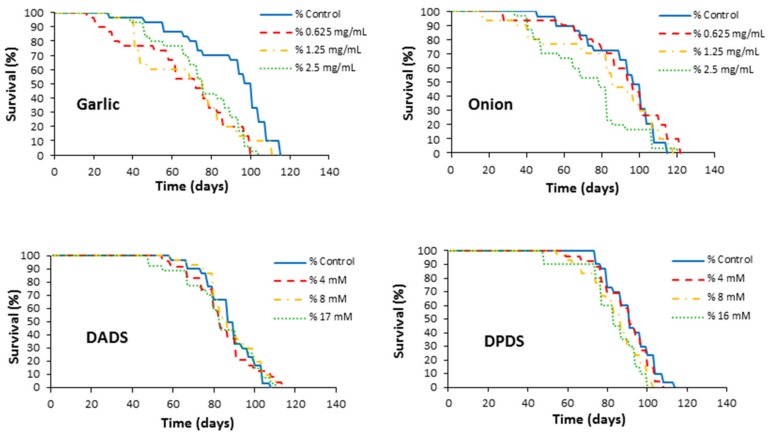
Effects of garlic, onion, DADS, and DPDS supplementation on the lifespan of *Drosophila melanogaster*.

Flies’ survival curves for all treatments are plotted in Figure 2. In general, all treatments induce lifespan maintenance. As shown in Table 2, only DPDS significantly decreased the lifespan at two supplementation levels (8 and 16 mM). DPDS and DADS significantly decreased the mean health span by 17% and 14%, respectively, only at the highest concentrations. It is noteworthy that there is an agreement between lifespan and health span significances of DPDS at 8 and 16 mM.

### 3.3. Cytotoxicity and Proapoptotic Assays in Leukemia Cells

**Figure 3:**
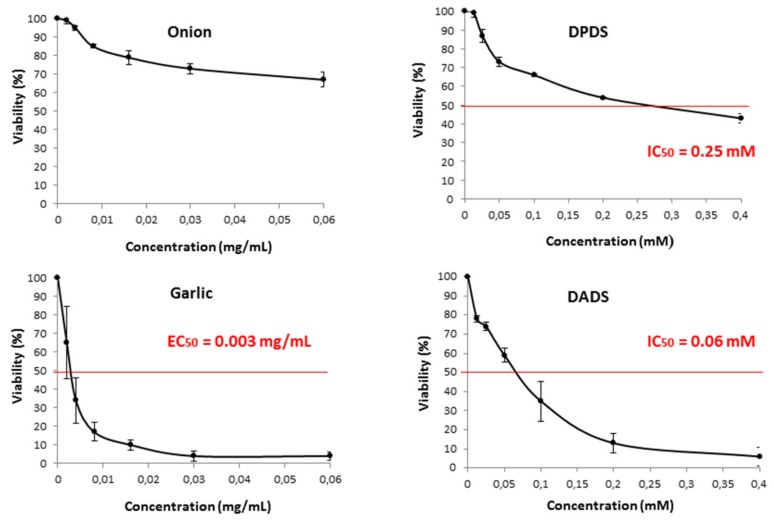
Viability of HL60 cells treated during 72 h with different concentrations of onion, garlic, and their respective organosulfur compound, DPDS, and DADS. Curves are plotted as mean percentages with respect to the control (three independent replicates). IC_50_: Inhibition concentration 50 for the tested organosulfur. EC_50_: effective concentration 50 for the tested extracts.

The cytotoxic effects of *Allium* vegetables and their distinctive compounds (DADS and DPDS) on the survival of HL60 cells are shown in Figure 3. Garlic and DADS exerted a cytotoxic effect on cell growth in a positive dose-dependent manner after 72 h of incubation, with EC_50_ of 0.003 mg/mL in the case of garlic and IC_50_ of 0.06 mM in the case of DADS. On the contrary, the effect observed in DPDS was smaller, with a high IC_50_ of 0.25 mM and it was absent in onion treatments in which the cytotoxic effect resulted only in a growth inhibition of 30% at the higher tested concentrations.

**Figure 4:**
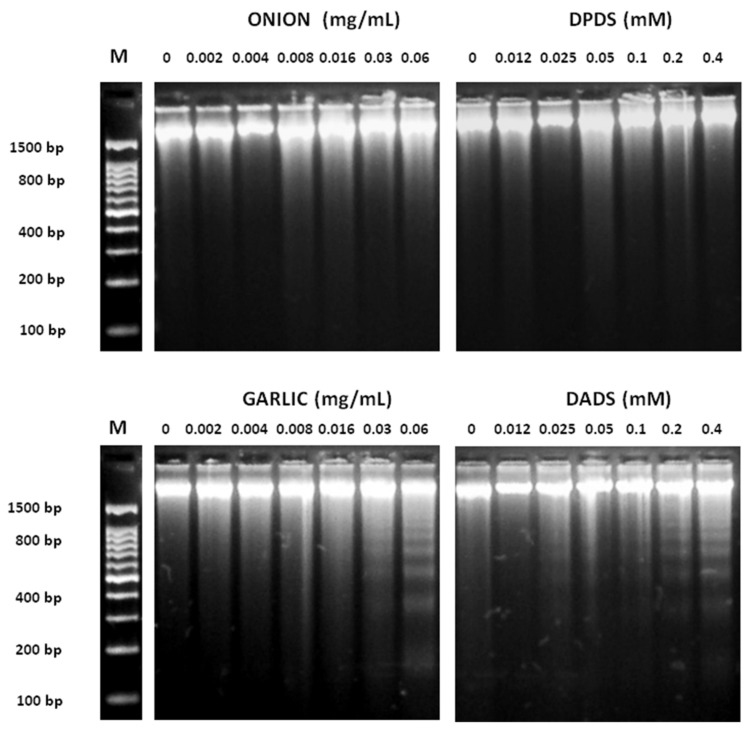
Inter-nucleosomal DNA fragmentation. HL-60 cells were exposed to various concentrations of onion, garlic, and their distinctive organo-sulfurs for 5 h. DNA was extracted from cells and was subject to 2% agarose gel electrophoresis at 50 V for 90 min. M: DNA size marker.

The results of proapoptotic effects of different concentrations of garlic, onion, DADS, and DPDS in HL60 cells measured as internucleosomic programmed fragmentation [66] are shown in Figure 4. DNA fragmentation was observed at high concentrations of garlic (0.03 and 0.06 mg/mL) and DADS (0.1, 0.2, and 0.4 mM). Nevertheless, any DNA inter-nucleosomal fragments were induced neither by onions nor by DPDS at the assayed concentrations.

### 3.4. DNA Single Strand Breaks

**Figure 5:**
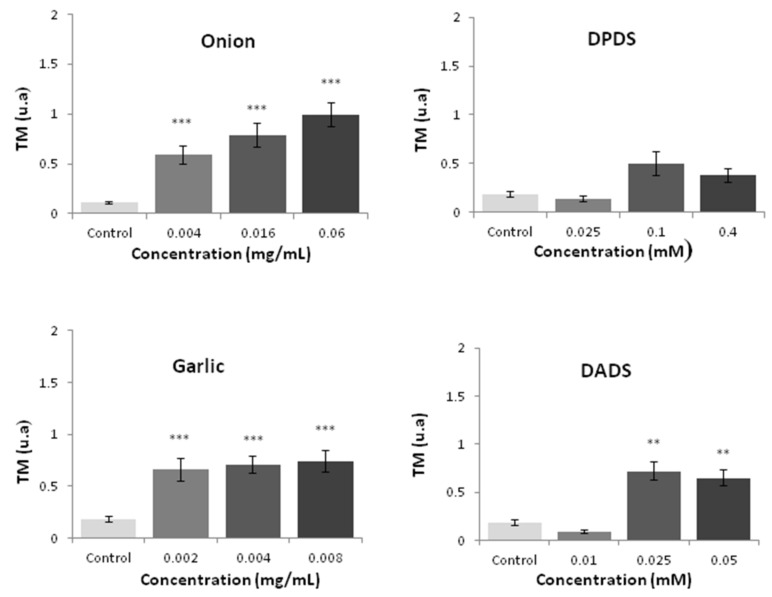
HL60 DNA integrity measured by the comet assay after 5 h of treatment with different concentrations of the tested compounds. Data are expressed as a TM parameter [54]. Statitiscal significance compared with a negative control: *** *p* ≤ 0.000 and ** *p* ≤ 0.01 for mean values of three independent replicates.

Both vegetables induced a significant (*p* ≤ 0.001) increase in the tail moment (TM) at all tested concentrations. On the contrary, only DADS (garlic with organosulfur) was able to induce a significant (*p* ≤ 0.01) increase of this parameter at 28 and 56 µM (Figure 5).

### 3.5. Methylation Status

**Figure 6:**
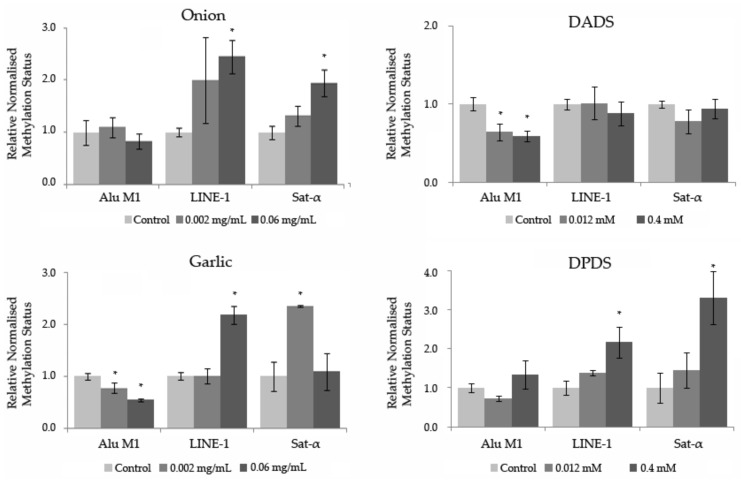
Methylation status of *Allium* sp. vegetables, DADS, and DPDS in HL-60 cells. Relative normalized expression data of each repetitive element (Alu M1, LINE-1, and Sat-α). Values represent the mean ± SE from three independent experiments. * *p* ≤ 0.05.

The relative normalized expression of three repetitive sequences (Alu M1, LINE-1, and Sat-α) studied in HL-60 cells treated with different concentrations of *Allium* sp. vegetables, DPDS, and DADS is shown in Figure 6. After one-way ANOVA and post hoc Tukey’s test, statistical results showed a significant hypermethylation level at LINE-1 and Sat- α repetitive sequences at the highest concentration tested of onion and DPDS. Moreover, garlic exhibited a significant hypermethylation status at the highest concentration tested in LINE-1 and at the lowest concentration tested in Sat-α sequences. Contrarily, a significant hypomethylation level of both assayed concentrations of DADS and garlic is shown in the Alu M1 repetitive sequences. The rest of the concentrations showed a similar methylation level to that of the normalized control.

## 4. Discussion

### 4.1. In Vivo Assessment of the Safety, Protection, and Lifespan Modulation

Garlic samples and onions have traditionally been used as food sources around the world across centuries likely due to their demonstrated particular flavor but also due to the health benefits, such as the prevention of cardiovascular diseases, cancer, and even aging [7]. Despite their popularity, the number of systematic, integrated, and multifocal studies assessing the genotoxic, antigenotoxic, and health span effects are scarce, and even less for assessing their distinctive organosulfur compounds (DADS and DPDS).

Our in vivo DNA stability studies (genotoxicity, antigenotoxicity, and longevity) were carried out using *D. melanogaster* flies. These organisms are widely used as a genetic animal model due to their homology with several mammal models in biological, physiological, and neurological traits [67,68]. It was demonstrated that more than 70% of human disease-causing genes have a functional homolog in this fly model [69]. Additionally, this particular model was also largely used to evaluate the genotoxicity of different biological compounds and molecules due to its accuracy, robustness, and reproducibility [70,71,72].

Carcinogen molecules and mutagenic properties should be taken into account and carefully evaluated in every complex mixture to be proposed for food. For this reason, genotoxic screening assays are considered as the first mandatory step, with the *Drosophila* wing spot test one of the most reliable methodologies to be employed as an ideal assay to evaluate biological products aimed to use in human and animal diets. To our knowledge, this is the first study to characterize the genotoxic effect of garlic, onion, and their two major and distinctive organosulfur constitutive molecules (DADS and DPDS, respectively) using the *D. melanogaster* animal model. Previous studies determined the lack of mutagenicity of these vegetables in a *Salmonella typhimurium* and in yeast models [73,74]. It has also been demonstrated that aqueous garlic extracts (5% *v/v*), fine garlic powder supplementation (7.5, 5, and 2.5 g/kg body weight), and fresh garlic bulb extracts (3, 6, and 12 mg/culture) were safe in vitro (cell lines) and non-animal models [74,75,76,77].

Our results for onion supplementation in the *Drosophila* model demonstrated a lack of genotoxicity, which validates previous reports obtained by Kulkarni et al. [78] in several *Salmonella* strains. In the same way, DPDS and DADS, which are the active principles in garlic and onion, were also non-genotoxic in our SMART trials. Despite the fact that onions are widely employed in the human diet, the number of genotoxicity studies carried out in DPDS are scarce [37,79]. Our study was the first to test the safety and protective effects of this compound using in vivo models. Nevertheless, previous reports assessing these particular molecules are controversial. For instance, Musk et al. demonstrated that DADS induced both chromosome aberrations and sister chromatid exchanges, characterized as genotoxic effects, at lower concentrations (below 0.07 mM) in a Chinese hamster ovary cell line [80]. However, this controversy could partially be explained due to methodological differences (in vivo vs. in vitro models) and the concentrations were tested. Controversial results are commonly found for a single molecule when it is tested in different assays and in vivo carcinogenic trials are needed.

One of the strategies for coping with the food and environmental genotoxic compounds is to identify effective antimutagens and anticarcinogens in order to increase man’s exposure to them as a way to decrease the cancer incidence [81]. This is the second step in the search of real nutraceutical substances. In our case, antigenotoxicity assays were conducted using hydrogen peroxide as a positive geno-toxicant model since this compound is able to induce somatic mutation and mitotic recombination in *D. melanogaster* [65], which affects the DNA integrity and stability.

Similar results to ours were reported on the desmutagenic activity of onions. Ethanolic extracts showed a strong inhibitory effect against NDBA in prokaryotes [32] and Welsh onion juice suppressed the mutagenic activity of benzo[a]pyrene (BaP) and 4-nitroquinoline 1-oxide (4QNO) and reduced the number of 2,4-dimethoxybenzaldehyde (DMBA)-induced chromosome aberrations in rats [82] while onion supplementation protected *D. melanogaster* against urethane-induced DNA damage [31]. All those reports validate our findings since onion supplementation reduced the mutagenic effects of H_2_O_2_ by as much as 65% in a dose-dependent manner. In the same way, DPDS showed des-mutagenic properties when it was tested as an individual molecule despite being at a lower extent when compared with the effect on onions. In this sense, DPDS strongly increased dimethyl nitrosamine (DMN) mutagenicity in *S. typhimurium* [37] and reduced NPYR/NDMA-induced oxidative DNA damage in HepG2 cells at 5 µM [38]. However, our study is the first one demonstrating that *Allium* vegetables have a protective role against H_2_O_2_ induced damage using the *D. melanogaster* model, which is a more adequate model widely used to extrapolate to mammals. This effect could be due to its well-known scavenging potential against free-radicals of their respective organosulfur compounds [18,83,84] since similar results were observed in the vegetables and simple molecule assessments.

The desmutagenic activity of garlic and different types of garlic extracts were previously described in several induced mutagenesis models. It was demonstrated that garlic and garlic water extracts protected against gamma-radiation and cyclophosphamide in mice [75,77,85]. In the same way, methanolic and ethanolic garlic extracts, even prepared by different processing methods (raw, grilled, and pickled), showed inhibitory activities on H_2_O_2_-induced DNA damage in human leukocytes [86] and reduced the chromosomal aberrations induced by DMBA in mice bone marrow [87]. In the same way, raw garlic methanolic extracts reduced the urethane mutagenicity in standard and high bioactivated *D. melanogaster* crosses [31]. In our experimental design, garlic clearly behaves as an anti-genotoxin, which could potentially be explained by the fact that concurrent experiments using DADS as simple molecule also inhibited the 95% of the H_2_O_2_-induced DNA damage. This desmutagenic property of DADS was previously proposed in several reports using different mutagenic substances such as (+)-anti-7β,8α-dihydroxy-9α,10α-oxy-7,8,9,10-tetrahydrobenzo[a]pyrene (BPDE), styrene oxide (SO), 4-NQO, aflatoxin B1 (AFB1), *N*-nitrosodimethylamine (NDMA), and 1-nitrosopyrrolidine (NPYR) [38,79,88].

Longevity assays are one of the most simple and efficient methodological approaches to evaluate the aging and anti-aging effects of simple compounds and complex mixtures on higher organisms. *D. melanogaster* is considered a very useful genetic model on aging research since its similarities with human metabolic pathways controlling nutrient uptake, storage, and metabolism [89,90]. In addition, this model has a short lifespan compared with similar in vivo models, which reduces the experimental periods.

To our knowledge, this is the first assessment on the effect of onions, DADS, and DPDS on the *D. melanogaster* lifespan and one of the few available assessing this effect in garlic samples [91]. These results support the hypothesis that individual organosulfur compounds can reduce longevity to some extent. These compounds could primarily be responsible for the apparent reduced viability observed in some cohort groups of flies. A similarity between the complete food and their distinctive compounds in the lifespan behavior is observed, although, in the case of vegetables, the lifespan is not significantly reduced when compared to the concurrent negative controls. Being onion and garlic complex mixtures of many individual molecules, the final outcome of such a complex trait longevity appears to be an additive combination of positive and negative synergic effects of the molecular components of vegetables with many of them phenolic and organosulfur, which are not included in the present study. Previous reports showed beneficial effects of garlic extracts on animal lifespan, including *D. melanogaster* and *C. elegans* [21]. Those differences could be due to the different tested samples being raw garlic in our study and garlic extracts in previous reports. In this sense, Prowse et al. demonstrated the insecticidal activity of garlic juices across several life stages of flies at a wide range of concentrations (0.25%–5%) in two dipteran pests (*Delia radicum* and *Musca domestica*) [92]. These results agree with the fact that similar but not significant effects on lifespan were caused by garlic, onion, DADS, and DPDS in our *D. melanogaster* experiments. It is noticeable that high doses were used for medicinal purposes in human acute treatments [93]. Thus, high dosages of garlic would not be advisable to be used in long-term chronic treatments due to the adverse effects that could be associated, even though nutraceuticals or dietary supplements include the bioactive compounds at higher doses than those used in this study.

### 4.2. In Vitro Assessment of the Cytotoxic, Clastogenic Activities and Methylation Status

Our results showed that only garlic and DADS have a strong cytotoxic effect and induce a clear DNA pro-apoptotic inter-nucleosomal fragmentation against HL60 cells. Previous reports demonstrated that garlic and DADS exerted a chemo-preventive effect through different pathways: (i) by increasing apoptosis and *Bcl-2* expression and decreasing p53 protein and *Bax* expression in lung cancer cells (NCI-H1299) [94], (ii) by increasing intracellular ROS in A549 cells [22], (iii) by inhibiting cell proliferation in CaCo-2 and HT-29 cells repressing histone deacetylase activity and histone hyperacetylation and increasing the p21(waf1/cip1) expression [95], and (iv) by inducing apoptosis by activating caspase-3 expression in HL60 cells [96]. In addition, Yang et al. observed that DADS supplementation (0.5, 10, and 25 µM) had a pro-apoptotic effect in COLO 205 cell line by inducing reactive oxygen species and caspase cascade [23]. On the contrary, the cytotoxic effect exerted by the onion and DPDS is relatively weak and their molecular mechanism is less clear. As an example, Sundaram and Milner [97] demonstrated that DPDS (100 µM) was an inefficient molecule to inhibit the cell growth and to induce programmed cell death in tumor cells (HCT-15). However, Wu et al. suggested that onion oil induces cell cycle arrest and apoptosis through ROS production in A549 cells [35]. It was also proposed that the carcinogenic inhibition mechanism of DADS is mediated through a modulation of the P450 cytochrome–dependent monooxygenases and/or the acceleration of carcinogen detoxification through phase II-enzymes upregulation [98,99]. In our case, the chemo-preventive properties of raw onion samples and DADS were weak despite the type of sample employed.

DNA inter-nucleosomal fragmentation was defined as one of the hallmarks of cellular apoptosis, even though it cannot be considered as a single criterion to assess the apoptotic cell death [100]. In order to determine the ability of our tested substances to induce DNA breaks in HL60 cells, we employed a single cell gel electrophoresis (comet) assay, which it is widely used to detect the apoptotic capability of mixtures and single compounds to induce DNA damage [101,102]. Currently, this procedure is being widely employed to evaluate the DNA stability in normal and carcinogenic cell lines against different substances, due to its robustness and reliability [103]. In this methodology, we employed the tail moment (TM) index, which is an accurate parameter to quantify the DNA migration and, thus, the DNA fragmentation status [54]. With this parameter, we differentiated apoptosis-induced from necrosis-induced DNA damage as follows: a TM ˃ 30 is considered to be an indicator of apoptosis and a TM between 5 and 30 a.u. (arbitrary units) is considered to be a necrotic process [104].

In this study, we determined for the first time the DNA-damage exerted by garlic, onion, DADS, and DPDS through the alkaline “comet assay” in HL60 leukemic cells in order to assess their potential anticarcinogenic effect. Our results (Figure 6) showed that onion and garlic induced DNA damage in HL60 by necrosis (short tails, TM < 2) being in concordance with our cytotoxic and DNA-fragmentation results. Similar results were observed in DADS and DPDS, but in a lower extent, which suggested the total absence of proapoptotic activity in the entire compound tested at the different assayed concentrations.

Our results with DPDS disagree with those obtained by Arranz and Haza [38], who showed that DPDS could act in a positive dose-dependent manner since the higher concentrations tested (˃5 µM) caused DNA damage in HepG2 cells (data not shown) by the comet assay. Arranz et al. assaying higher concentrations of DADS (˃5 µM), showed DNA damage in HepG2 cells in the alkaline comet assay [38]. However, controversial results were also reported by Belloir et al., which suggests that DADS was not genotoxic at concentrations between 5 to 100 µM in the same in vitro model [105].

Despite a non-significant relationship shown in the methylation status of *Allium* sp. vegetables, DPDS, and DADS in the three repetitive sequences studied, a general tendency to hypermethylate the genomic randomized-distributed sequences of the HL-60 cells (LINE-1 and Sat-α) is shown.

Based on our knowledge, no previous studies about the in vitro effects that *Allium* sp. vegetables, DPDS, and DADS have in the methylation status of three repetitive sequences of treated HL-60 tumor cells. Taking into account that methylation of the repetitive sequences is understood as an important genomic protective mechanism [62,106], high concentrations of *Alliium* sp. vegetables and DPDS could have positive effects on tumor cells, that could be an interesting chemo-preventive effect. On the other hand, negative effects on tumor cells are related to garlic and DADS in the short repetitive element studied (Alu M1).

## 5. Conclusions

To sum up, our experimental results provide the evidence that (i) garlic, onion, DADS, and DPDS are safe substances, which exert an antigenotoxic effect against oxidative mutagens in a dose-dependent manner. (ii) The decrease of lifespan induced in the *Drosophila* animal model by DPDS at the highest concentrations could be a signal that the long-term consumption of complex mixtures is safe only at low concentrations. (iii) Garlic exerted a clear chemo-preventive effect, with its distinctive organosulfur DADS as the most likely cause of such activities. (iv). The slight cytotoxic effect of onions is probably mediated by a non-apoptotic mechanism. Overall, this study could be a baseline for further supplementary studies to clarify the cell death pathway induced by garlic and DADS. (v) A general increase of the methylation status in LINE-1 and Sat-α repetitive sequences of HL-60 treated cells are shown in onions, garlic, and DPDS, which is related to a genomic protective mechanism.

## Figures and Tables

**Table 1:** Genotoxicity and antigenotoxicity results obtained in the SMART test when flies were fed with different concentrations of onions, garlic, and organosulfur DPDS and DADS in single and combined treatments.

Clones Per Wings (Number of Spots) ^(1)^						
Compounds	*N*	Small Spots (1–2 cells)	Large Spots (˃2 cells)	Twin Spots	Total Spots	Mann-Whitney Test ^(3)^
**Controls**						
H_2_O	40	0.10 (4) ^(2)^	0	0	0.10 (4)	
H_2_O_2_ (0.12M)	40	0.30 (12)	0.05 (2)	0.02 (1)	0.37 (15) +	Ω
**Onion (mg/mL)**						
0.625	40	0.17 (7)	0	0	0.17 (7) i	Δ
5	38	0.08 (3)	0.03 (1)	0	0.10 (4) i	Δ
0.625 + H_2_O_2_	40	0.20 (8)	0	0.05 (2)	0.25 (10) λ	Ω
5 + H_2_O_2_	38	0.08 (3)	0	0.03 (1)	0.11 (4) β	
**Garlic (mg/mL)**						
0.625	40	0.07 (3)	0	0	0.07 (3) i	Δ
5	40	0.05 (2)	0	0	0.05 (2) i	Δ
0.625 + H_2_O_2_	40	0.27 (11)	0	0.02 (1)	0.30 (12) λ	Δ
5 + H_2_O_2_	40	0.15 (6)	0	0	0.15 (6) β	
**DPDS (mM)**						
4	40	0.22 (9)	0.02 (1)	0.02 (1)	0.27 (11) i	Δ
33	40	0.07 (3)	0.07 (3)	0	0.15 (6) i	Δ
4 + H_2_O_2_	40	0.20 (8)	0.05 (2)	0	0.25 (10) λ	Ω
33 + H_2_O_2_	40	0.17 (7)	0.02 (1)	0	0.20 (8) λ	Ω
**DADS (mM)**						
4	40	0.15 (6)	0	0	0.15 (6) i	Δ
34	26	0.04 (1)	0	0	0.04 (1) i	Δ
4 + H_2_O_2_	40	0.20 (8)	0.02 (1)	0	0.22 (9) λ	Ω
34 + H_2_O_2_	40	0.02 (1)	0	0	0.02 (1) β	

^1^ Statistical diagnosis according to Frei and Würgler [48]. + (positive) and i (inconclusive) versus negative control. β (significantly different) and λ (inconclusive) versus positive control. m: multiplication factor. Kastenbaum-Bowman Test without Bonferroni correction and probability levels α = β = 0.05. ^2^ Number of spots or clones in parentheses. ^3^ Inconclusive and positive results were resolved using the Mann-Whitney U-test. Delta marker (∆) means no differences between the treatments and the concurrent control. Ohm marker (Ω) means differences between the treatments and the concurrent control.

**Table 2:** Effects of the tested compounds at different concentrations on the *Drosophila melanogaster* mean lifespan and health span.

	Mean Lifespan (Days)	Mean Lifespan Difference (%) ^a^	Health-Span (75th Percentile) (Days)	Health-Span Difference (%) ^a^
**Onion (mg/mL)**				
Control	92.24 ± 3.58	0	76.00 ± 12.63	0
0.625	95.77 ± 3.45	4	83.00 ± 5.04	9
1.25	92.83 ± 3.36	1	83.00 ± 5.08	9
2.5	81.92 ± 4.98	−11	65.00 ± 13.34	−11
**Garlic (mg/mL)**				
Control	81.25 ± 4.57	0	51.14 ± 4.31	0
0.625	79.51 ± 3.30	−2	58.83 ± 1.76	15
1.25	76.21 ± 4.46	−6	47.29 ± 3.73	−7
2.5	77.68 ± 3.35	−4	53.57 ± 3.48	5
**DPDS (mM)**				
Control	91.63 ± 2.06	0	77.37 ± 1.05	0
4	89.58 ± 2.39	−2	73.00 ± 2.80	−6
8	84.23 ± 2.38*	−8	67.12 ± 2.69 **	−13
16	82.25 ± 3.23*	−10	64.20 ± 6.63 *	−17
**DADS (mM)**				
Control	88.00 ± 2.26	0	73.11 ± 2.40	0
4	84.64 ± 3.01	−4	68.00 ± 3.34	−7
8	88.83 ± 2.36	1	75.22 ± 2.50	3
17	86.21 ± 3.27	−2	62.87 ± 3.96 *	−14

^a^ Difference between treated flies and the concurrent negative control (water) in percentage. Positive results indicate that lifespan was increased and negative results indicate that lifespan was decreased. Statistical significance: * = *p* ≤ 0.05, ** = *p* ≤ 0.01 (log-Mantel-Cox test).
